# Secondary Metabolites from Polar Organisms

**DOI:** 10.3390/md15030028

**Published:** 2017-02-23

**Authors:** Yuan Tian, Yan-Ling Li, Feng-Chun Zhao

**Affiliations:** 1 College of Life Science, Taishan Medical University, Taian 271016, Shandong, China; lingl816@163.com; 2 College of Life Science, Shandong Agricultural University, Taian 271018, Shandong, China; zhaofengchun@126.com

**Keywords:** natural products, secondary metabolism, structure elucidation, biological activity, biosynthesis, chemical synthesis

## Abstract

Polar organisms have been found to develop unique defences against the extreme environment environment, leading to the biosynthesis of novel molecules with diverse bioactivities. This review covers the 219 novel natural products described since 2001, from the Arctic and the Antarctic microoganisms, lichen, moss and marine faunas. The structures of the new compounds and details of the source organism, along with any relevant biological activities are presented. Where reported, synthetic and biosynthetic studies on the polar metabolites have also been included.

## 1. Introduction

Organisms from special ecosystems such as the polar regions are a rich source of various chemical scaffolds and novel natural products with promising bioactivities. Polar regions, which refer to the Arctic, the Antarctic and their subregions, are remote and challenging areas on the earth. To survive under the constant influence of low temperatures, strong winds, low nutrient and high UV radiation or combinations of these factors [1], polar organisms require a diverse array of biochemical and physiological adaptations that are essential for survival. These adaptations are often accompanied by modifications to both gene regulation and metabolic pathways, increasing the possibility of finding unique functional metabolites of pharmaceutical importance.

Polar regions are complex ecosystems that harbor diverse groups of fauna and microorganisms including bacteria, actinomycetes and fungi. Physiological adaptations have enabled psychrophilic organisms to thrive in the polar regions, especially microorganisms which are high in number and usually uncharacterized [2,3,4,5]. However, when compared to the large number of polar microorganisms which have been reported, very few have been screened for the production of interesting secondary metabolites. The advent of modern techniques provides the opportunity to find novel metabolites.

From 2001 to 2016, a vast amount of new biological natural compounds with various activities, such as anti-bacteria, anti-tumor, anti-virus and so on, have been isolated from polar organisms including microorganisms, lichen, moss, bryozoans, cnidarians, echinoderms, molluscs, sponges and tunicates. Natural products from the Arctic or the Antarctic organisms have been the subject of several review articles. In 2007, Lebar et al. reviewed the studies on structure and bioactivity of cold-water marine natural products, including many polar examples [6]. In 2009, Wilson and Brimble reviewed molecules derived from the extremes of life, including some polar examples [7]. In 2011, advances in the chemistry and bioactivity of arctic sponge were reviewed by Hamann and his co-workers [8]. In 2013, Liu et al. reviewed a number of new secondary metabolites with various activities derived from both Antarcitc and Arctic organisms [9], while in 2014, Skropeta and Wei published a review on natural products isolated from deep-sea sources, which included some polar organisms [10]. Moreover, Blunt and his co-workers published periodical reviews on the characteristics of various marine natural products with some polar examples [11,12,13].

However, comprehensive reviews of natural products from polar regions were rare; therefore, we describe here the source, chemistry, and biology of the newly discovered biomolecules from the polar organisms. We also summarize the chemical synthesis and the biosynthetic relationship of metabolites. The Metabolites Name Index in combination with the Source Index, the Biological Activity Index and the References on isolation in the accompanying tables, will help understand the fascinating chemistry and biology of natural products derived from polar organisms.

## 2. Microorganisms

The microbial diversity of polar environments is a fertile ground for new bioactive compounds, genes, proteins, microorganisms and other products with potential for commercial use [14].

### 2.1. Unicellular Bacteria

**Figure 1:**
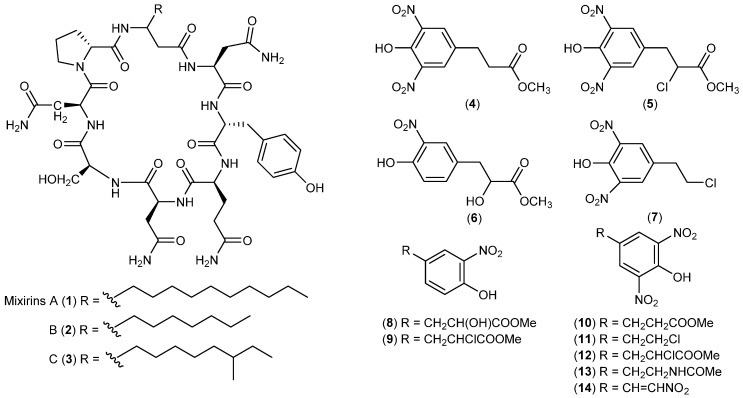
Secondary metabolites derived from the Arctic bacteria (compounds **1**–**14**).

The culture broth of the marine bacterium *Bacillus* sp., isolated from the sea mud near the Arctic pole, was found to yield three new cyclic acylpeptides named as mixirins A (**1**), B (**2**) and C (**3**) (Figure 1) [15]. All of the three compounds were found to display significant cytotoxicity against human colon tumor cells (HCT-116) with half maximal inhibitory concentration (IC_50_) values of 0.68, 1.6, 1.3 μg/mL, respectively.

Four new aromatic nitro compounds (**4**–**7**) (Figure 1) along with fifteen known ones were reported from the *Salegentibacter* strain T436, isolated from a bottom section of a sea ice floe collected from the Arctic Ocean. The new natural products showed weak antimicrobial and cytotoxic activities [16]. Further study of the same bacterium isolate yielded another seven new aromatic nitro compounds (**8**–**14**) (Figure 1) [17].

**Figure 2:**
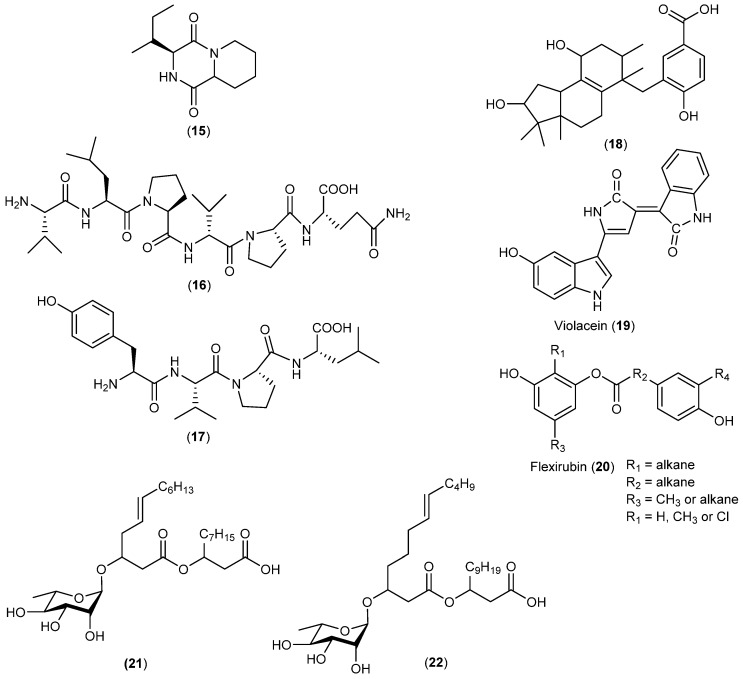
Secondary metabolites derived from the Antarctic bacteria (compounds **15**–**22**).

A novel diketopiperazine, named cyclo-(d-pipecolinyl-l-isoleucine) (**15**) (Figure 2), and two new linear peptides (**16**, **17**) (Figure 2), along with seven known diketopiperazines were isolated from the cell-free culture supernatant of the Antarctic psychrophilic bacterium *Pseudoalteromonas haloplanktis* TAC125 [18]. Peptide **17** and a known phenyl-containing diketopiperazine showed free radical scavenging properties, with the phenyl group essential for activity.

From the Antarctic cyanobacterium *Nostoc* CCC 537, an antibacterial lead molecule (**18**) (Figure 2) was obtained. Compound **18** exhibited antibacterial activity against two Gram positive pathogenic strains and seven Gram negative strains including three multi-drug resistant strains of *Escherichia coli*, with minimal inhibition concentration (MIC) values in the range of 0.5–16.0 μg/mL [19].

Two pigments named violacein (**19**) and flexirubin (**20**) (Figure 2) were isolated from two Antarctic bacterial strains. The two compounds displayed antibacterial activities against some mycobacteria with low MIC values (ranging from 2.6 to 34.4 μg/mL), and might be valuable natural lead compounds for new antimycobacterial drugs used for tuberculosis chemotherapy [20].

Bioassay-guided purification of Antarctic strain *Pseudomonas* BNT1 extracts produced three rhamnolipids including two new ones (**21**, **22**). Compound **21** was effective against the tested human pathogens strains *Burkholderia cepacia*, *B. metallica*, *B. seminalis*, *B. latens* and *Staphylococcus aureus* with low MIC and minimun bacteriocidal concentration (MBC) values, while compound **22** only had moderate antimicrobial effect against *S. aureus* with an MBC value of 100 μg/mL [21].

### 2.2. Actinomycetes

**Figure 3:**
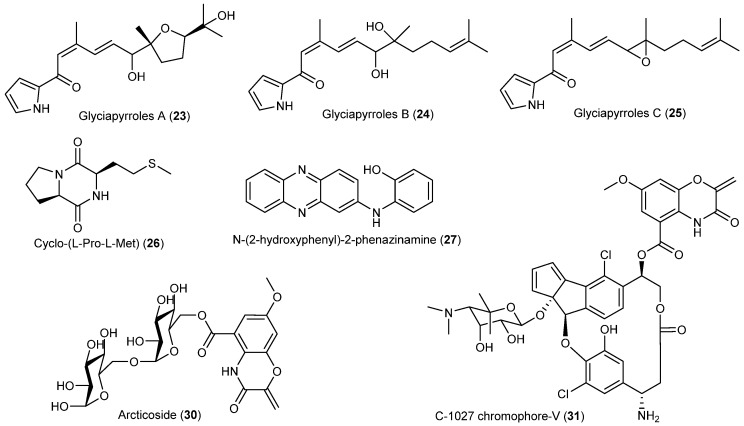
Secondary metabolites derived from the Arctic actinomyces (compounds **23**–**27**, **30**, **31**).

In this century, actinomyces derived from polar regions have yielded an array of interesting new metabolites. Three new pyrrolosesquiterpenes, glyciapyrroles A (**23**), B (**24**), and C (**25**) (Figure 3), along with three known ones, iketopiperazines cyclo(leucyl-prolyl), cyclo(isoleucyl-prolyl), and cyclo(phenylalanyl-prolyl), were isolated from the *Streptomyces* sp. NPS008187 originating from a marine sediment collected in Alaska near the Arctic [22].

The Arctic seaweed-associated actinomycete *Nocardiopsis* sp. 03N67 was found to produce a rare bioactive diketopiperazine, cyclo-(l-Pro-l-Met) (**26**) (Figure 3). It inhibited tumor necrosis factor-α (TNF-α)-induced tube formation and invasion at 10 μM, a concentration at which no cytotoxicity was observed. Anti-angiogenesis activity against human umbilical vein endothelial cells (HUVECs) of compound **26** is an encouraging bioprobe to develop new anticancer therapeutics from such type of small molecules in near future [23].

The marine actinomycete *Nocardia dassonvillei* BM-17, obtained from a sediment sample collected in the Arctic Ocean, has furnished a new secondary metabolite, *N*-(2-hydroxyphenyl)-2-phenazinamine (**27**) (Figure 3), and six known antibiotics. The new compound showed weak antifungal activity against *Candida albicans*, with a MIC value of 64 μg/mL and low cancer cell cytotoxicity against HepG2, A549, HCT-116 and COC1 cells, with IC_50_ values of 40.33, 38.53, 27.82 and 28.11 μg/mL, respectively [24].

**Scheme 1:**
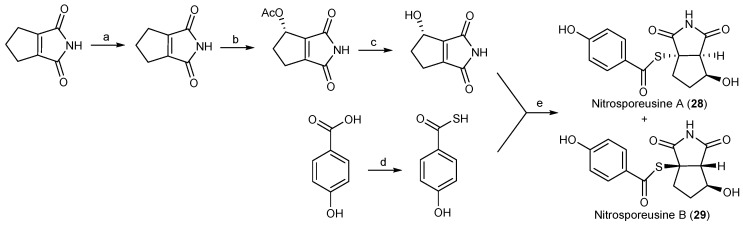
Synthesis of natural products nitrosporeusines A (**28**) and B (**29**). Reagents and conditions: (**a**) SeO_2_, 1,4-dioxane, microwave, 110 °C, 61%; (**b**) Amano PS lipase, THF, CH_2_=CHOAc, 38%; (**c**) Amano PS lipase, phosphate buffer, 92%; (**d**) Lawesson's reagent, acetonitrile, microwave, 100 °C, 53%; (**e**) H_2_O, room temperature (rt), 65%.

Chemical examination from the Arctic actinomycete *Streptomyces nitrosporeus* CQT14-24 resulted in the isolation of two new alkaloids, named as nitrosporeusines A (**28**) and B (**29**) (Scheme 1), with an unprecedented skeleton containing benzenecarbothioc cyclopenta[c]pyrrole-1,3-dione. Both **28** and **29** showed inhibitory activity against the influenza WSN virus (H1N1) in Madin–Darby canine kidney (MDCK) cells with the dose of 50 μM. In an in vitro plaque reduction assay, **29** exhibited dose-dependent reduction of the production of the viral progeny which was produced by the infected MDCK cells with influenza A/WSN/33 virus. The half effective concentration (EC_50_) value of **29** for the inhibition of viral plaque formation was quite comparable to that of the positive control oseltamivir phosphate (Osv-P) [25]. Their biological activities have attracted interest to synthesize these compounds. Efficient stereoselective synthesis of the natural enantiomer of nitrosporeusines A and B was performed by Reddy’s groups. An overall five-step process starting from 5,6-dihydrocyclopenta[*c*]pyrrole-1,3(2*H*,4*H*)-dione and p-hydroxybenzoic acid is summarized in Scheme 1 [26].

Two new secondary metabolites, arcticoside (**30**) and C-1027 chromophore-V (**31**) (Figure 3), were isolated along with three kown compounds, C-1027 chromophore-III, fijiolides A and B from the culture of an Arctic marine actinomycete *Streptomyces* strain. Compounds **30** and **31** inhibited *Candida albicans* isocitrate lyase, an enzyme that plays an important role in the pathogenicity of *C. albicans.* Furthermore, **31** exhibited significant cytotoxicity against breast carcinoma MDA-MB231 cells and colorectal carcinoma HCT-116 cells, with IC_50_ values of 0.9 and 2.7 μM, respectively [27].

**Figure 4:**
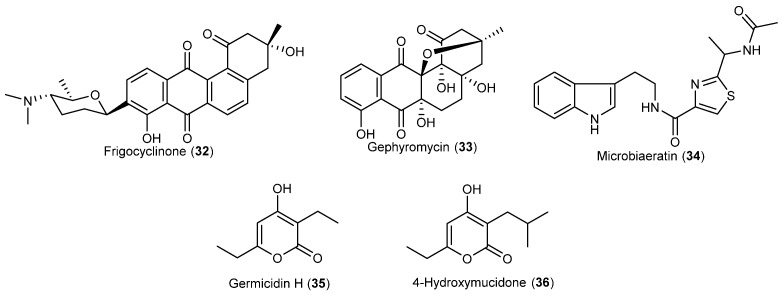
Secondary metabolites derived from the Antarctic actinomyces (compounds **32**–**36**).

A *Streptomyces griseus* strain NTK 97, recovered from an Antarctic terrestrial sample, yielded a new angucyclinone antibiotic frigocyclinone (**32**) (Figure 4), consisting of a tetrangomycin moiety attached through a C-glycosidic linkage with the aminodeoxysugar ossamine. Frigocyclinone revealed good inhibitory activities against *Bacillus subtilis* and *Staphylococcus aureus* [28]. Another Antarctic *Streptomyces griseus* strain NTK14 was shown to contain the novel-type angucyclinone gephyromycin (**33**) (Figure 4), and two known compounds, fridamycin E and dehydrorabelomycin. Gephyromycin, with an unprecedented intramolecular ether bridge, displayed glutaminergic activity (agonist) towards neuronal cells. In addition, **33** exhibited no acute cytostatic activities, and the lack of cytotoxicity made its neuroprotective properties even more valuable [29].

A new sulphur-containing natural alkaloid named microbiaeratin (**34**) (Figure 4) was characterized, together with the known bacillamide from the culture of *Microbispora aerata* strain IMBAS-11A, isolated from the Antarctic Livingston Island. A low antiproliferative and cytotoxic effects of 34 was determined with L-929 mouse fibroblast cells, K-562 human leukemia cells and HeLa human cervix carcinoma cells [30].

The *Nocardiopsis* sp. SCSIO KS107 was isolated from Antarctic seashore sediment. Fermentation and isolation of this strain provided two new α-pyrones germicidin H (**35**), 4-hydroxymucidone (**36**), and a known compound 7-hydroxymucidone. Only the known compound showed antibacterial activity against *Micrococcus luteus* with an MIC value of 16 μg/mL [31].

### 2.3. Fungi

**Figure 5:**
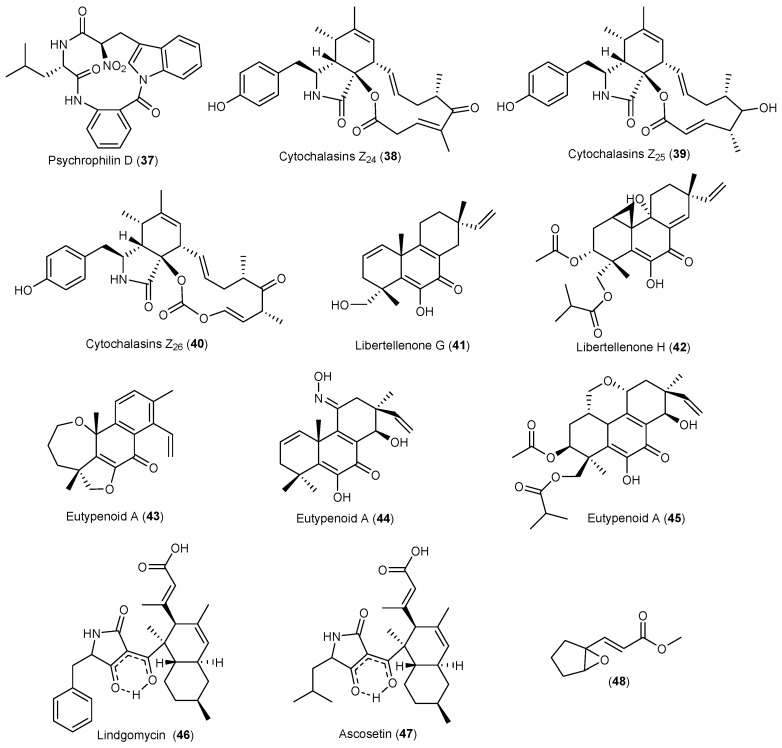
Secondary metabolites derived from the Arctic fungi (compounds **37**–**48**).

The rapid growth and ability to metabolize a wide variety of substrates have enabled fungi to become the predominant components of the microorganisms in polar regions. The psychrotolerant fungus *Penicillium algidum*, collected from soil under a *Ribes* sp. in Greenland near the Arctic, yielded the new cyclic nitropeptide, psychrophilin D (**37**) (Figure 5), together with two known cyclic peptides, cycloaspeptide A and cycloaspeptide D. The compounds were tested in antimicrobial, antiviral, anticancer and antiplasmodial assays. Psychrophilin D exhibited a moderate activity with half infective dose (ID_50_) value of 10.1 μg/mL in the P388 murine leukaemia cell assay. Cycloaspeptide A and D exhibited moderate activity (IC_50_ = 3.5 and 4.7 μg/mL, respectively) against *Plasmodium falciparum* [32].

Three new cytochalasins Z_24_, Z_25_, Z_26_ (**38**–**40**) (Figure 5) and one known compound, scoparasin B, were isolated from the Arctic fungus *Eutypella* sp. D-1. These compounds were evaluated for cytotoxic activities against several human tumor cell lines. Among them, compound **38** showed moderate cytotoxicity toward human breast cancer MCF-7 cell line with IC_50_ value of 9.33 mΜ [33]. Further investigation of *Eutypella* sp. D-1 led to the discovery of two new diterpenes, libertellenone G (**41**) and libertellenone H (**42**) (Figure 5), together with two known pimarane diterpenes. Compound **41** exhibited antibacterial activity against *Escherichia coli*, *Bacillus subtilis* and *Staphylococcus aureus*. Compound **42** showed slight cytotoxicity toward most cell lines, with half-maximal inhibitory concentration values ranging from 3.31 to 44.1 μM. In addition, the cytotoxicity of **42** is most likely dependent on the presence of its cyclopropane ring as deduced from the inactivity of other similar compounds [34]. Recently, three pimarane diterpenoids, Eutypenoids A–C (**43**–**45**), were also isolated from the culture of *Eutypella* (*E*.) sp. D-1. Using a ConA-induced splenocyte proliferation model, compound 44 exhibited potent immunosuppressive activities [35].

An unusual polyketide with a new carbon skeleton, lindgomycin (**46**) (Figure 5) [36], and the recently described ascosetin (**47**) (Figure 5) [37] were extracted from different Lindgomycetaceae strains, which were isolated from an Arctic sponge. Both the compounds exhibited strong antibiotic activities against the clinically relevant Gram-positive bacteria (including methicillin-resistant *Staphylococcus aureus*) and human pathogenic yeast *Candida albicans*.

*Trichoderma polysporum* strain OPU1571, recovered from a moss, *Sanionia uncinata*, growing in the high arctic wetlands on Spitsbergen Island, Svalbard, Norway, yielded eleven compounds, including a new one (**48**) (Figure 5). The in vitro investigation suggested that compound **48** showed a concentration-dependent growth-inhibitory effect on snow rot pathogen *Pythium iwayamai* at 5 days [38].

**Figure 6:**
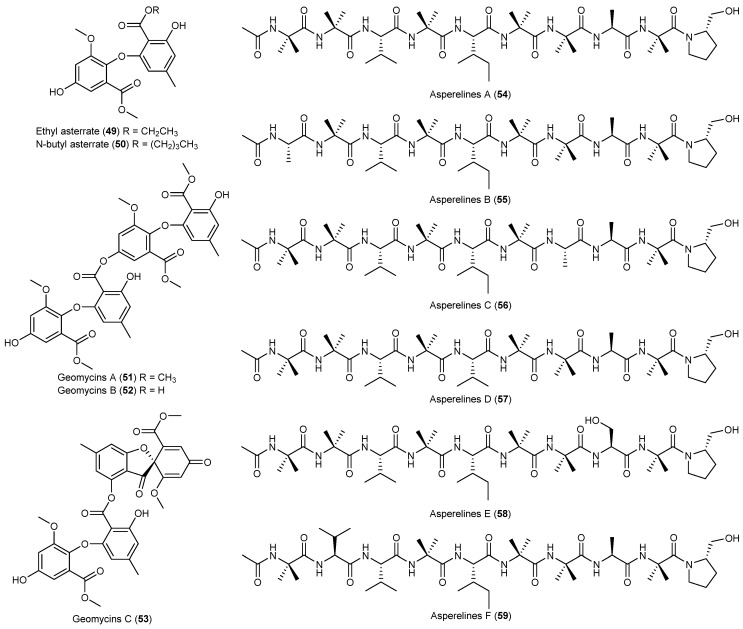
Secondary metabolites derived from the Antarctic fungi (compounds **49**–**59**).

Fermentation of the Antarctic ascomycete fungus *Geomyces* sp. yielded five new asterric acid derivatives, ethyl asterrate (**49**), n-butyl asterrate (**50**), and geomycins A–C (**51**–**53**) (Figure 6). The new metabolites were tested for their antibacterial and antifungal activities. Geomycin B (**52**) showed significant antifungal activity against *Aspergillus fumigatus* ATCC 10894, with IC_50_/MIC values of 0.86/29.5 μM (the positive control fluconazole showed IC_50_/MIC values of 7.35/163.4 μM). Geomycin C (**53**) displayed moderate antimicrobial activities against the Gram-positive bacteria (*Staphylococcus aureus* ATCC 6538 and *Streptococcus pneumoniae* CGMCC 1.1692) and Gram-negative bacterium (*Escherichia coli* CGMCC 1.2340) [39].

Chemical investigation of the marine-derived fungus *Trichoderma asperellum*, collected from the sediment of the Antarctic Penguin Island, resulted in the isolation of six new peptaibols named asperelines A–F (**54**–**59**) (Figure 6), which are characterized by an acetylated N-terminus and a C-terminus containing an uncommon prolinol residue. The compounds were tested against fungi and bacteria, but they showed only weak inhibitory activity toward the early blight pathogen *Alternaria solani*, the rice blast *Pyricularia oryzae*, and the bacteria *Staphylococcus aureus* and *Escherichia coli* [40]. Further study on the same fungus strain determined thirty-eight short peptaibols, including thirty-two new compounds namely asperelines G–Z_13_ [41].

**Figure 7:**
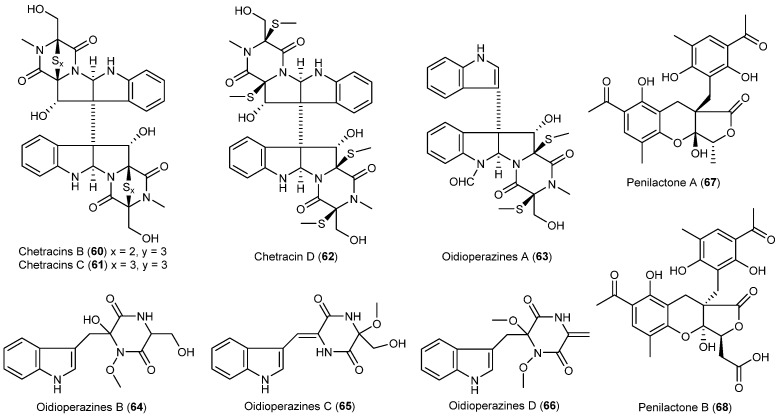
Secondary metabolites derived from the Antarctic fungi (compounds **60**–**68**).

Two new epipolythiodioxopiperazines, named chetracins B (**60**) and C (**61**), and five new diketopiperazines, named chetracin D (**62**) and oidioperazines A–D (**63**–**66**) (Figure 7), were obtained from the Antarctic fungus *Oidiodendron truncatum* GW3-13. An in vitro 3-(4, 5-dimethylthiazol-2-yl) 2, 5-diphenyl tetrazolium bromide (MTT) cytotoxicity assay revealed potent biological activity for 60 in the nanomolar range against a panel of five human cancer lines, HCT-8, BEL-7402, BGC-823, A-549 and A-2780. New metabolites **61** and **62** displayed significant cytotoxicity at a micromolar concentration, whereas **63**–**66** showed no significant cytotoxicity at 10 μM. Comparison of the bioactivity data suggested that the sulfide bridge was a determinant factor for their cytotoxicity, while the number of sulfur atoms in the bridge did not seem to influence activity [42].

**Scheme 2:**
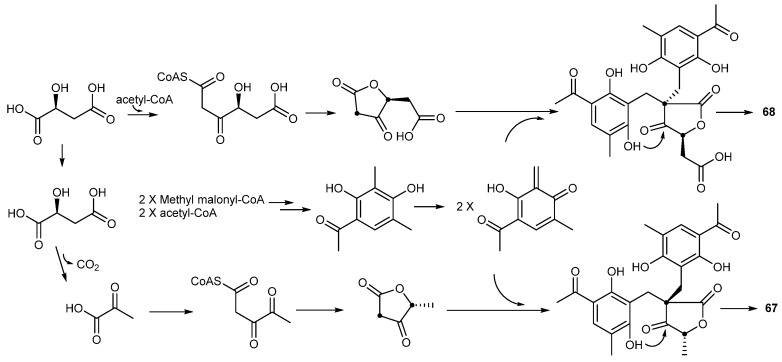
Proposed biosynthesis of penilactones A (**67**) and B (**68**).

**Scheme 3:**
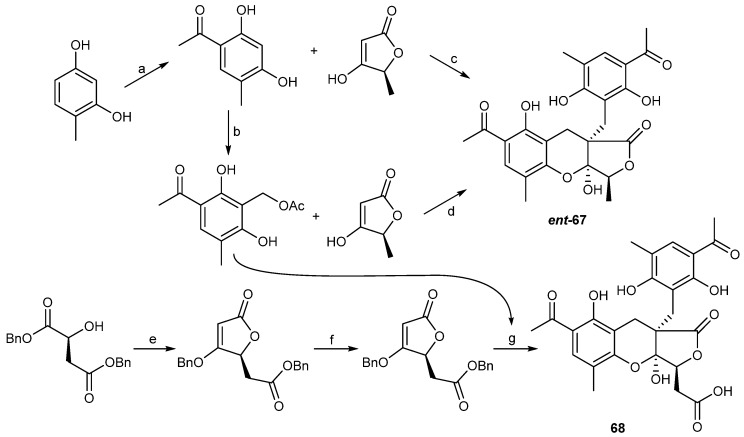
Synthesis of *ent*-penilactone A (*ent-***67**) and penilactone B (**68**). Reagents and conditions: (**a**) AcOH, BF_3_.Et_2_O, 90 °C, 71%; (**b**) HCHO, NaOAc, AcOH, 80 °C, 75%; (**c**) HCHO, NaOAc, AcOH, 90 °C, then 110 °C, 46%; (**d**) toluene, 110 °C, 93%; (**e**) Ph_3_P=C=C=O, toluene, 110 °C, 52%; (**f**) H_2_, PD/C, MeOH, rt, 99%; (**g**) dioxane, 110 °C, 86%.

In 2012, two highly oxygenated polyketides, penilactones A and B (**67** and **68**) (Figure 7) of related structure but opposite absolute stereochemistry, were isolated from the Antarctic deep-sea derived fungus *Penicillium crustosum* PRB-2. The nuclear factor-κB (NF-κB) inhibitory activities of **67** and **68** were tested by means of transient transfection and reporter gene expression assay, and only **67** showed weak activity with an inhibitory rate of 40% at a concentration of 10 μM. A plausible biosynthetic pathway for **67** and **68** was proposed as shown in Scheme 2 [43]. The penilactones contain a new carbon skeleton formed from two 3,5-dimethyl-2,4-diol-acetophenone units and a γ-butyrolactone moiety, and have been prepared by a biomimetic synthesis reported the following year as shown in Scheme 3 [44].

**Scheme 4:**
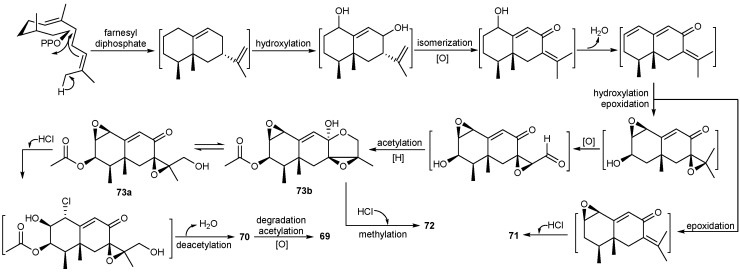
Proposed biogenetic network for compounds **69**–**73**.

**Figure 8:**
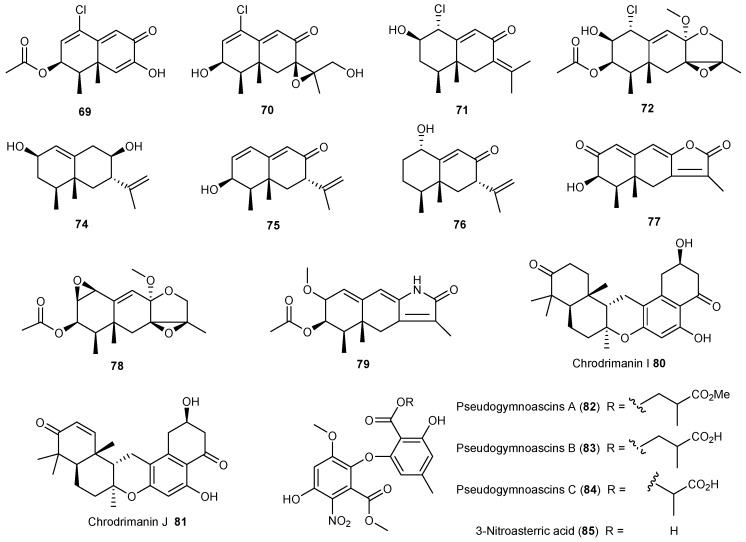
Secondary metabolites derived from the Antarctic fungi (compounds **69**–**72**, **74**–**85**).

A new chloro-trinoreremophilane sesquiterpene (**69**) (Figure 8), three new chlorinated eremophilane sesquiterpenes (**70**–**72**) (Figure 8), together with a known compound, eremofortine C (**73**) (Scheme 4), were isolated from the Antarctic deep-sea derived fungus, *Penicillium* sp. PR19N-1 in 2013. Compound **69** showed moderate cytotoxic activity against HL-60 and A549 cancer cell lines. In addition, the plausible metabolic network of these isolated products was proposed as demonstrated in Scheme 4 [45]. Further investigation of this strain yielded five new eremophilane-type sesquiterpenes (**74**–**78**) and a new rare lactam-type eremophilane (**79**) (Figure 8). Their cytotoxities against HL-60 and A-549 human cancer cell lines were valuated, and **78** was the most active one with IC_50_ value of 5.2 μM against the A-549 cells [46].

Two new meroterpenoids, named chrodrimanins I and J (**80** and **81**) (Figure 8), together with five known biosynthetically related chrodrimanins, were isolated from the culture of the Antarctic moss-derived fungus *Penicillium funiculosum* GWT2-24. Distinguished from all of the reported chrodrimanins, compounds **80** and **81** possess a unique cyclohexanone (E ring) instead of a δ-lactone ring. However, only the known compounds exhibited inhibitory activities against influenza virus A (H1N1) [47].

Fermentation of a *Pseudogymnoascus* sp. fungus isolated from an Antarctic marine sponge, yielded four new nitroasterric acid derivatives, pseudogymnoascins A–C (**82**–**84**) and 3-nitroasterric acid (**85**) (Figure 8), along with two known compounds questin and pyriculamide. These compounds are the first nitro derivatives of the known fungal metabolite asterric acid [48].

**Figure 9:**
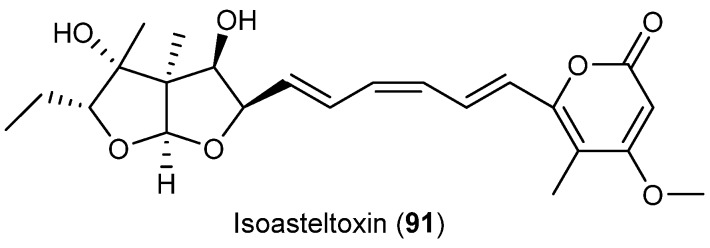
The structure of isoasteltoxin (**91**) derived from the Antarctic fungus *Aspergillus ochraceopetaliformis* SCSIO 05702.

**Scheme 5:**
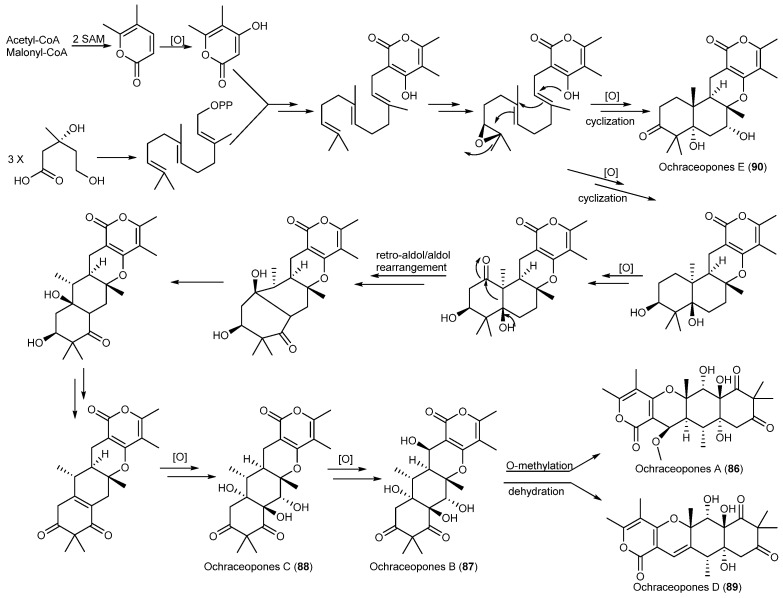
Postulated biogenetic pathway for ochraceopones A–E (**86**–**90**).

Five new highly oxygenated α-pyrone merosesquiterpenoids, ochraceopones A–E (**86**–**90**) (Scheme 5), together with one new double bond isomer of asteltoxin, isoasteltoxin (**91**) (Figure 9), and two known asteltoxin derivatives, asteltoxin and asteltoxin B, were isolated from the Antarctic soil-derived fungus *Aspergillus ochraceopetaliformis* SCSIO 05702. Ochraceopones A–D (**86**–**89**) were the first examples of α-pyrone merosesquiterpenoids possessing a linear tetracyclic carbon skeleton. All the isolated compounds were tested for their antiviral, cytotoxic, antibacterial, and antitubercular activities. Among the new compounds, **86** and **91** exhibited promising antiviral activities against the H1N1 and H3N2 influenza viruses. In addition, a possible biosynthetic pathway for ochraceopones A–E was proposed in Scheme 5 [49].

## 3. Lichen

Lichens are symbiotic associations of fungi and algae and/or cyanobacteria that produce unique characteristic secondary metabolites by comparison to those of higher plants. Several species of lichens have been used for various remedies in folk medicine since ancient time and variety of biologically active lichen metabolites, including antimycobacterial, antiviral, antioxidant and antiherbivore properties, have been described [50,51]. Antarctic lichens may have evolved unique secondary metabolites to help in surviving the extreme environment in which they live [52,53,54].

**Figure 10:**
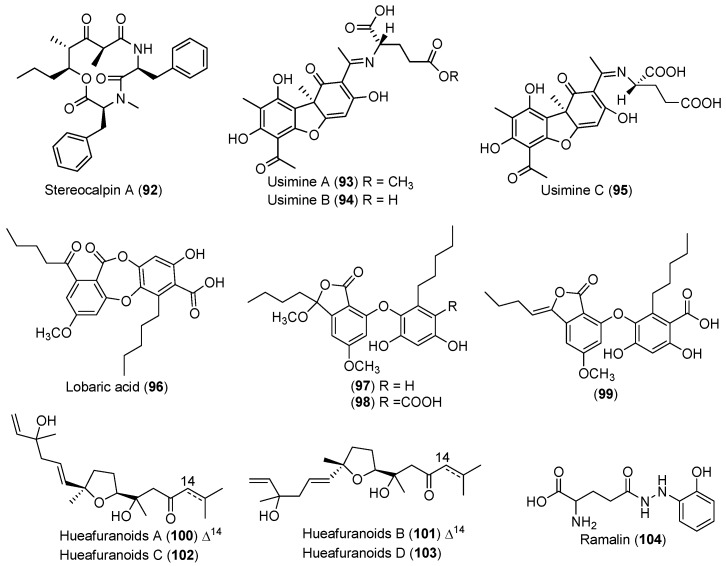
Secondary metabolites derived from the Antarctic lichen (compounds **92**–**104**).

Professor Oh’s group has made great efforts in discovery of new metabolites from the Antarctic lichens. From the MeOH extract of the Antarctic lichen *Stereocaulon alpinum*, a new cyclic depsipeptide stereocalpin A (**92**) (Figure 10) [55], together with three new usnic acid derivatives usimines A–C (**93**–**95**) (Figure 10) [56], were isolated by various chromatographic methods. Compound **92** incorporated an unprecedented 5-hydroxy-2,4-dimethyl-3-oxo-octanoic acid in the structure, and showed marginal levels of cytotoxicity against three human tumor cell lines, HT-29, B16/F10 and HepG2. In addition, compounds **92**–**95** moderately inhibited the activity of protein tyrosine phosphatase 1B (PTP1B) in a dose-dependent manner. Inhibition of PTP1B is predicted to be an excellent, novel therapy to target type 2 diabetes and obesity [57]. Furher investigation of this Antarctic lichen recovered seven phenolic lichen metabolites. Among the compounds, the depsidone-type compound, lobaric acid (**96**) (Figure 10) and two pseudodepsidone-type compounds, **97** and **98** (Figure 10), exhibited potent inhibitory activity against PTP1B with low IC50 values in a non-competitive manner [58]. In 2013, a new pseudodepsidone-type metabolite, lobastin (**99**) (Figure 10), with antioxidant and antibacterial activity was also reported from this strain [59].

Four new diterpene furanoids, hueafuranoids A–D (**100**–**103**) (Figure 10) have been isolated from the MeOH extract of the Antarctic lichen *Huea* sp. Compound **100** showed inhibitory activity against therapeutically targeted protein, PTP1B with an IC50 value of 13.9 μM [60].

From the Antarctic lichen *Ramalina terebrata*, the novel compound ramalin (**104**) (Figure 10) was isolated. The experimental data showed that ramalin displayed potent antioxidant activity and can be a strong therapeutic candidate for controlling oxidative stress in cells [61].

## 4. Mosses

**Figure 11:**
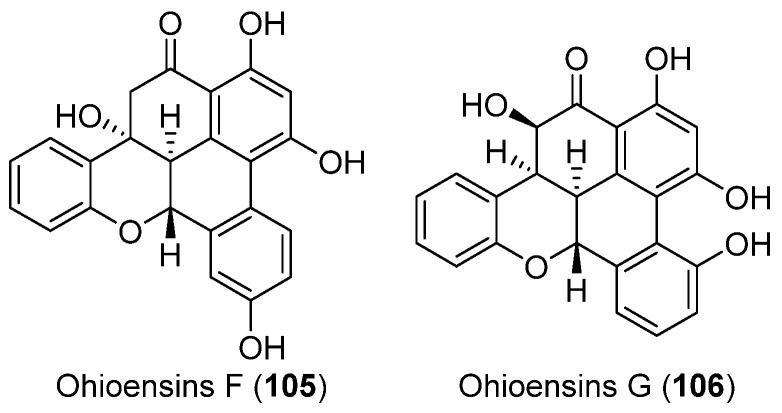
Secondary metabolites derived from the Antarctic moss (compounds **105**, **106**).

Mosses represent a relatively untapped natural source to be explored for new bioactive metabolites. Chemical studies of Antarctic moss *Polytrichastrum alpinum* led to the isolation of two new benzonaphthoxanthenones, ohioensins F and G (**105** and **106**) (Figure 11), along with two known compounds ohioensins A and C. All of the four compounds showed potent inhibitory activity against therapeutically targeted protein tyrosine phosphatase 1B (PTP1B). Kinetic analysis of PTP1B inhibition by ohioensin F (**105**) suggested that benzonaphthoxanthenones inhibited PTP1B activity in a non-competitive manner [62].

## 5. Bryozoans

**Figure 12:**
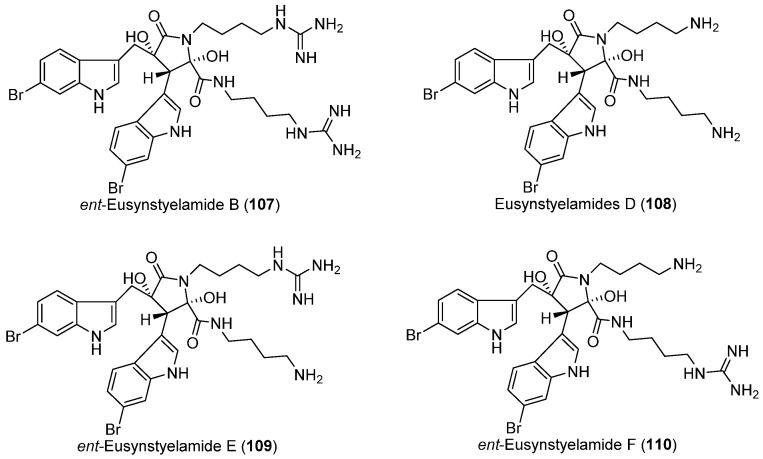
Secondary metabolites derived from the Arctic bryozoan (compounds **107**–**110**).

Bryozoans (moss animals and lace corals) have yielded a significant number of bioactive metabolites and have been reviewed elsewhere [63]. However, there is only a single report on secondary metabolites from a polar bryozoan. In 2011, an investigation into the chemistry of the Arctic bryozoan *Tegella* cf. *spitzbergensis* resulted in the isolation and structural determination of *ent*-eusynstyelamide B (**107**) and three new derivatives, eusynstyelamides D–F (**108**–**110**) (Figure 12) [64]. *Ent*-eusynstyelamide B (**107**) is the enantiomer of the known brominated tryptophan metabolite eusynstyelamide B [65]. Antimicrobial activities were reported for **107**–**110**, with MIC values as low as 6.25 μg/mL for **107** and **110** against *Staphylococcus aureus*. Eusynstyelamides were generally more active against Gram-positive bacteria than Gram-negative bacteria [64].

## 6. Cnidarians

**Figure 13:**
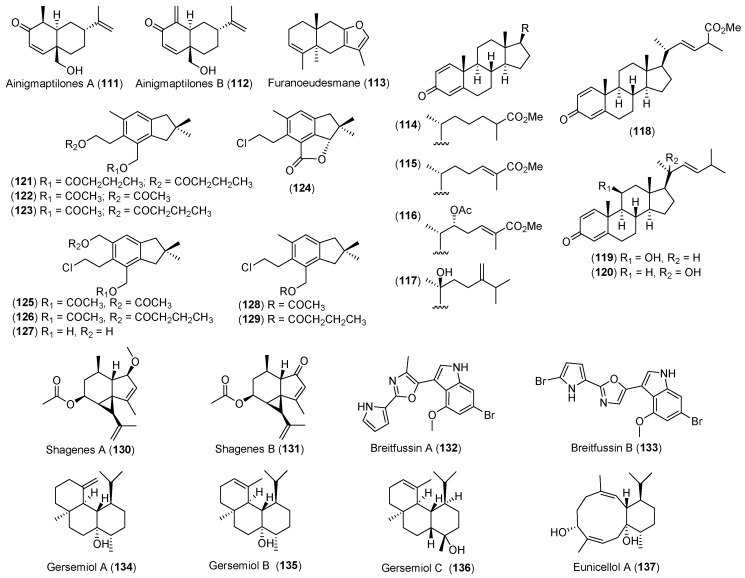
Secondary metabolites derived from the polar cnidarians (compounds **111**–**137**).

Cnidarians are the second largest source (after sponges) of new marine natural products reported each year, with a predominance of terpenoids [11,12,13] and are also well represented in the polar regions. In 2003, fractionation of the bioactive extract from the chemically defended Antarctic gorgonian coral *Ainigmaptilon antarcticus* yielded two sesquiterpenes, ainigmaptilones A (**111**) and B (**112**) (Figure 13). Ainigmaptilone A has broad spectrum bioactivity toward sympatric predatory and fouling organisms, including antibiotic activity [66].

The ethereal extract of another Antarctic gorgonian *Dasystenella acanthina* was found to contain three main nonpolar and relatively transient sesquiterpene metabolites, including a new compound, furanoeudesmane (**113**) (Figure 13). Compound **113** was toxic at 46 μM in a *Gambusia affinis* ichthyotoxicity test. According to this result, an involvement of these molecules in the defensive mechanisms of the animal could be suggested [67].

Seven new steroids, compounds **114**–**120** (Figure 13), were isolated from the Antarctic octocoral *Anthomastus bathyproctus*. The in vitro cytotoxicity has been tested against three human tumor cell lines MDA-MB-231 (breast adenocarcinoma), A-549 (lung carcinoma), and HT-29 (colon adenocarcinoma). Compounds **115**–**118** displayed weak activity as inhibitors of cell growth [68].

Chemical investigation of the lipophilic extract of the Antarctic soft coral *Alcyonium grandis* led to the finding of nine unreported sesquiterpenoids, compounds **121**–**129** (Figure 13) [69]. These molecules are members of the illudalane class and in particular belong to the group of alcyopterosins. Similar illudalanes have been isolated from the sub-Antartic deep sea soft coral *Alcyonium paessleri* [70]. Repellency experiments conducted using the omnivorous Antarctic sea star *Odontaster validus* revealed a strong activity in the lipophilic extract of *A. grandi*s against predation [69]. The total synthesis of some members of the alcyopterosin family has been reported already by many research groups [71,72,73,74,75].

More recently, the isolation and characterization of two new tricyclic sesquiterpenoids, shagenes A (**130**) and B (**131**) (Figure 13) were presented. The two compounds were isolated from an undescribed soft coral collected from the Scotia Arc in the Southern Ocean. Exploration of the bioactivity found that shagenes A was active against the visceral leishmaniasis causing parasite, *Leishmania donovani* (IC_50_ value = 5 μM), with no cytotoxicity against the mammalian host [76].

**Scheme 6:**
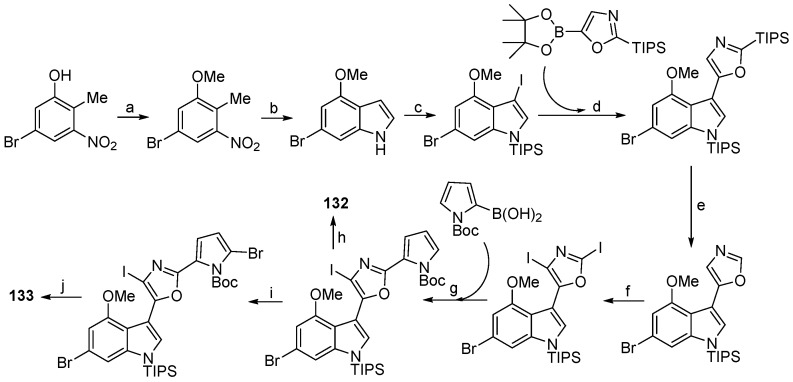
Synthesis of Breitfussin A (**132**) and B (**133**). Reagents and conditions: (**a**) MeI, Cs_2_CO_3_, DMF, rt, 82%; (**b**) Dimethylformamide dimethyl acetal (DMFDMA), Pyrrolidine, DMF, then Zn, AcOH/H2O, 80 °C, 61%; (**c**) Iodine chloride, Pyridine (ICl, Py), CH_2_Cl_2_, then NaH, Three isopropyl silicon alkyl chloride (TIPSCl), THF, 81%; (**d**) 1,1′-Bis(diphenylphosphino)ferrocene]dichloropalladium (Pd(dppf)Cl_2_), K_3_PO_4_, Toluene/H_2_O, 80 °C; (**e**) 10% aq HCl, THF, 0 °C, 89%; (**f**) Lithium hexamethyldisilazide (LiHMDS), −78 °C, then I_2_, −78 °C, 15%; (**g**) Pd(dppf)Cl_2_, Cs_2_CO_3_, Dioxane/H_2_O, rt, 61%; (**h**) Trimethylsilyl trifluoromethanesulfonate (TMSOTf), Et_3_N, CH_2_Cl_2_, 0 °C to rt, then Tetrabutylammonium fluoride (TBAF), THF, 0 °C, 64%; (**i**) *N*-Bromosuccinimide (NBS), THF, −78 °C to rt, 57%; (**j**) Trifluoroacetic acid (TFA), CH_2_Cl_2_, 0 °C to rt, then TBAF, THF, 0 °C, 67%.

In 2012, two halogenated natural products breitfussin A (**132**) and breitfussin B (**133**) (Figure 13) were isolated from the Arctic hydrozoan *Thuiria breitfussi* collected at Bjørnøya (Bear Island) [77]. Recently, Hedberg and co-workers synthesized breitfussins A and B firstly using Suzuki coupling reactions to join the heteroaromatic rings, thus confirming the assigned structure of these natural products (Scheme 6) [78]. Soon after, a conceptually distinct synthesis of breitfussin B through late-stage bromination of the breitfussin core was reported by Khan and Chen [79].

From the Arctic soft coral *Gersemia fruticosa*, three new diterpenes named gersemiols A–C (**134**–**136**) together with a new eunicellane diterpene, eunicellol A (**137**), have been obtained. All compounds were tested for their antimicrobial activity against several bacteria and fungi. Only eunicellol A was found to exhibit moderate anti-bacterial activity against methicillin resistant *Staphylococcus aureus* (MRSA) with MIC value of 24–48 μg/mL [80].

## 7. Echinoderms

**Figure 14:**
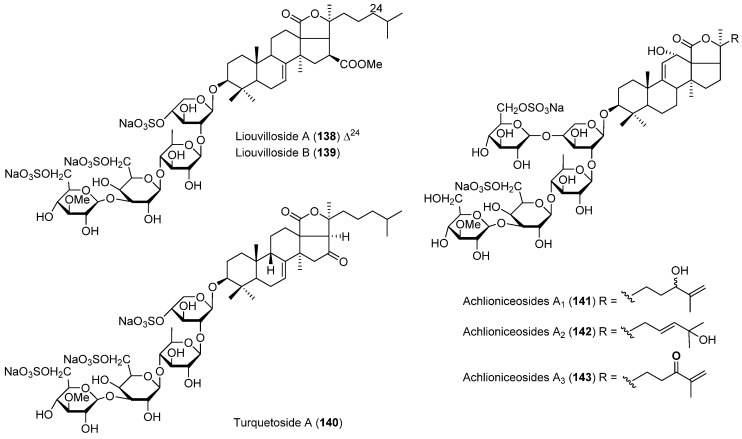
Secondary metabolites derived from the Antarctic sea cucumbers (compounds **138**–**143**).

Echinoderms are well known producers of bioactive glycosylated metabolites [11,12,13], and many new natural products have been described from the polar examples. In 2001, two new trisulfated triterpene glycosides, liouvillosides A (**138**) and B (**139**) (Figure 14), were isolated from the Antarctic sea cucumber *Staurocucumis liouvillei*. Liouvillosides A and B are two new examples of a small number of trisulfated triterpene glycosides from sea cucumbers belonging to the family Cucumariidae. Both glycosides were found to be virucidal against herpes simplex virus type 1 (HSV-1) at concentrations below 10 μg/mL [81]. A novel triterpene holostane disulfated tetrasaccharide olygoglycoside, turquetoside A (**140**) (Figure 14), having a rare terminal 3-*O*-methyl-d-quinovose, was isolated from the Antarctic sea cucumber *Staurocucumis turqueti* [82]. The occurrence of 3-*O*-methyl-d-quinovose in triterpene glycosides in *S. turqueti* and in the congeneric Antarctic sea cucumber *S. liouvillei* suggests that the presence of this sugar in carbohydrate chains of triterpene glycosides is a taxonomical character of the genus *Staurocucumis* [81,82].

Subsequently, another three new triterpene glycosides, achlioniceosides A1 (**141**), A2 (**142**), and A3 (**143**) (Figure 14), were obtained from the Antarctic sea cucumber *Achlionice violaecuspidata*. Glycosides **141**–**143** are the first triterpene glycosides isolated from a sea cucumber belonging to the order Elasipodida [83].

## 8. Molluscs

**Figure 15:**
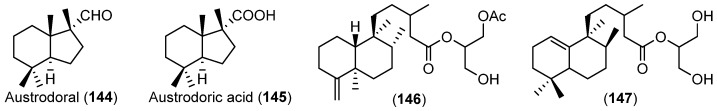
Secondary metabolites derived from the Antarctic nudibranch *Austrodoris kerguelenensis* (compounds **144**–**147**).

**Scheme 7:**
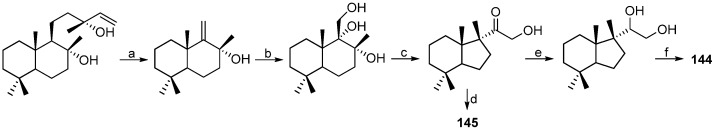
Synthesis of austrodoral (**144**) and austrodoric acid (**145**). Reagents and conditions: (**a**) four steps, 59% [87,88]; (**b**) OsO_4_, H_2_O, *t-*BuOH, trimethylamine *N*-oxide, pyridine, reflux, 24 h, 87%; (**c**) BF_3_·OEt_2_, CH_2_Cl_2_, 0 °C to rt, 20 min, 95%; (**d**) NaBH_4_, EtOH, rt, 15 min, 97%; (**e**) Pb(OAc)_4_, CH_2_Cl_2_, rt, 45 min, 92%; (**f**) NaIO_4_, *t*-BuOH–H_2_O, reflux, 12 h, 91%.

The nudibranch *Austrodoris kerguelenensis* is distributed widely around the Antarctic coast and continental shelves. In 2003, two unprecedented nor-sesquiterpenes, austrodoral (**144**) and its oxidised derivative austrodoric acid (**145**) (Figure 15), were isolated from the skin of the marine dorid *A. kerguelenensis*, collected in the Antarctic. A role of stress-metabolites could be suggested for these compounds [84]. A short and efficient synthesis of **144** and **145** was reported as shown in Scheme 7 [85]. Further chemical study of this Antarctic nudibranch yielded two novel 2-monoacylglycerols (**146**) and (**147**) (Figure 15), along with two known 1,2-diacyl glyceryl esters [86].

**Figure 16:**
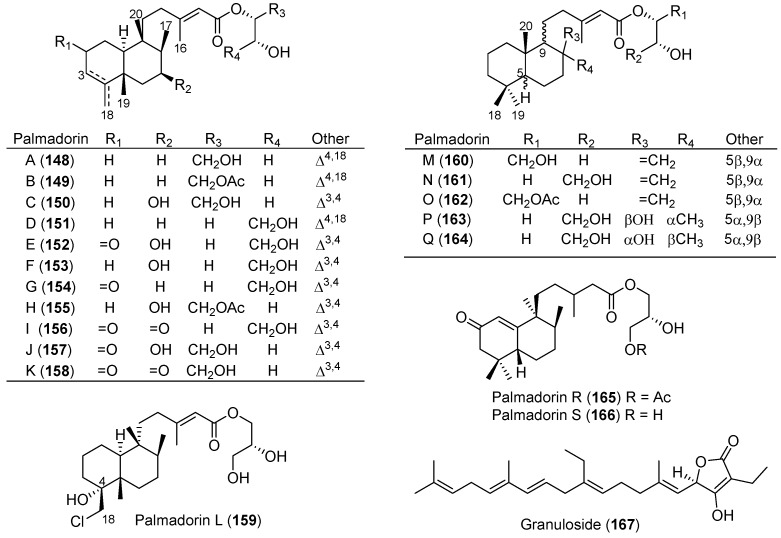
Secondary metabolites derived from the Antarctic nudibranch (compounds **148**–**167**).

Investigation of the nudibranch *A. kerguelenensis* collected near the Antarctic Peninsula resulted in the isolation of three new diterpenes, palmadorins A–C (**148**–**150**) (Figure 16) [89]. Detailed investigation of this Antarctic nudibranch led to the discovery of a diverse suite of new diterpenoid glyceride esters, palmadorins D–S (**151**–**166**) (Figure 16), including one (palmadorin L) that is the first halogenated diterpene from this well-studied nudibranch. Palmadorin A (**148**), B (**149**), D (**151**), M (**160**), N (**161**), and O (**162**) inhibit human erythroleukemia (HEL) cells with low micromolar IC_50_, and palmadorin M inhibits Jak2, STAT5, and Erk1/2 activation in HEL cells and causes apoptosis at 5 mM [90].

Recently, a new homosesterterpene named granuloside (**167**) (Figure 16), was characterized from the Antarctic nudibranch *Charcotia granulosa*. Granuloside was the first linear homosesterterpene skeleton ever reported and, despite the low molecular complexity, its chemical structure poses many questions about its biogenesis and origin in the nudibranch [91].

## 9. Sponges

Marine sponges are the largest source of new marine natural products reported annually [11,12,13] and have provided a rich array of biologically important compounds [92]. There are a large number of studies on sponges from warm or tropical waters, whereas little is known about the chemistry of sponges from the Arctic or Antarctic waters. Actually, polar species of marine sponges can also be a rich source of biologically and structurally interesting molecules.

**Figure 17:**
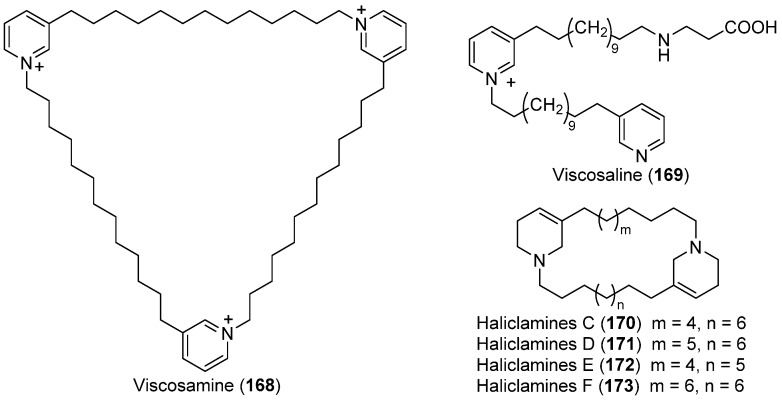
Secondary metabolites derived from the Arctic sponge *Haliclona viscosa* (compounds **168**–**173**).

Pyridinium alkaloids are widely distributed in marine sponges of different genera. In 2003, chemical investigation of the Arctic sponge *Haliclona viscosa* led to the isolation of a new trimeric 3-alkyl pyridinium alkaloid, viscosamine (**168**) (Figure 17) [93]. Further investigation of this sponge yielded one new 3-alkyl pyridinium alkaloid, viscosaline (**169**) (Figure 17) [94], and two new 3-alkyltetrahy-dropyridine alkaloids, haliclamines C (**170**) and D (**171**) (Figure 17) [95]. In 2009, new haliclamines E (**172**) and F (**173**) (Figure 17) were subsequently obtained from this Arctic sponge [96]. Compound **169** showed activity in the feeding deterrence assay against the amphipod *Anonyx nugax* and the starfish *Asterias rubens* from the North Sea [97]. Compounds **169** and **170** showed a strong inhibition against two bacterial strains isolated from the vicinity of the sponge [95,97]. The synthesis of the Arctic sponge alkaloids were also achieved by two groups [97,98].

**Scheme 8:**
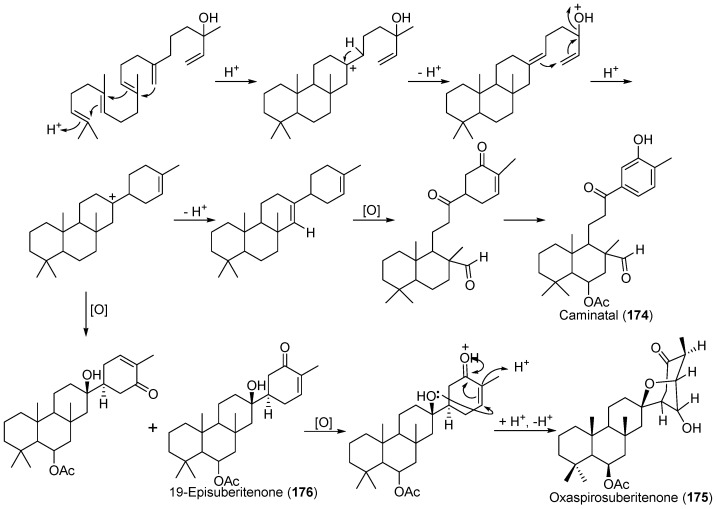
Possible biogenesis of caminatal (**174**), oxaspirosuberitenone (**175**) and 19-episuberitenone (**176**).

A new sesterterpene, caminatal (**174**), and two novel sesterterpenes, oxaspirosuberitenone (**175**) and 19-episuberitenone (**176**) (Scheme 8) were obtained from the Antarctic sponge *Suberites caminatus* [99,100]. Their possible biogenesis were proposed as shown in Scheme 8.

**Figure 18:**
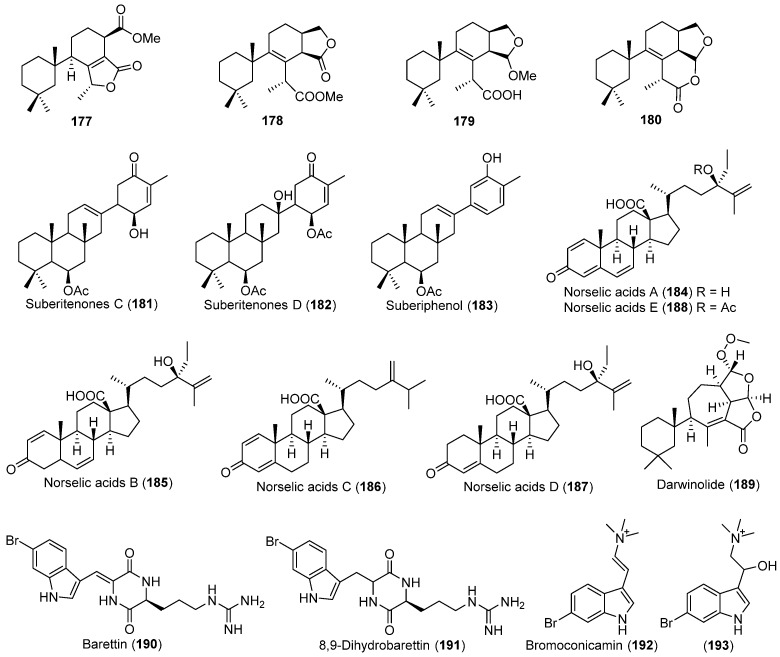
Secondary metabolites derived from the Antarctic sponges (compounds **177**–**189**) and the Arctic sponge (compounds **190**–**193**).

Four new diterpenoids, **177**–**180** (Figure 18) were isolated from the Antarctic sponge *Dendrilla membranosa*. Compound **177** was a nor-diterpene gracilane skeleton derivative, while **178**–**180** are C-20 aplysulphurane-type diterpenes [101].

Three new sesterterpenes of the suberitane class, suberitenones C (**181**) and D (**182**) and suberiphenol (**183**) (Figure 18), were isolated from the sponge *Suberites* sp. collected from Antarctica. Unfortunately, the new compounds were neither cytotoxic against the human leukemia K562 cell-line nor antimicrobial against *B. subtilis*, *C. albicans*, *E. coli* and *S. aureus* [102].

Five new steroids, norselic acids A–E (**184**–**188**) (Figure 18), were isolated from the sponge *Crella* sp. collected in Antarctica. Norselic acid A displayed antibiotic activity against methicillin-resistant *Staphylococcus aureus*, methicillin-sensitive *S. aureus*, vancomycin-resistant *Enterococcus faecium* and *Candida albicans*, and reduced consumption of food pellets by sympatric mesograzers. Compounds **184**–**188** were also active against the Leishmania parasite with low micromolar activity [103].

Darwinolide (**189**), a novel spongian diterpene, was characterized from the Antarctic sponge *Dendrilla membranosa*. Darwinolide displayed antibiofilm activity against MRSA with no mammalian cytotoxicity, and may present a suitable scaffold for the development of novel antibiofilm agents to treat drug resistant bacterial infections [104].

Barettin (**190**), 8,9-dihydrobarettin (**191**), bromoconicamin (**192**) and a novel brominated marine indole (**193**) (Figure 18) were isolated from the sponge *Geodia barretti* collected off the Norwegian coast. The compounds were evaluated as inhibitors of electric eel acetylcholinesterase. Compounds **190** and **191** displayed significant inhibition of the enzyme, **192** was less potent against acetylcholinesterase, and **193** was inactive. Based on the inhibitory activity, a library of 22 simplified synthetic analogs was designed and prepared to probe the role of the brominated indole. From the structure–activity investigation, it was shown that the brominated indole motif is not sufficient to generate a high acetylcholinesterase inhibitory activity, even when combined with natural cationic ligands for the acetylcholinesterase active site. The four natural compounds were also analysed for butyrylcholinesterase inhibitory activity and shown to display comparable activities [105].

## 10. Tunicates

Tunicates comprise >2800 species and have yielded a diverse array of bioactive metabolites [10,106], including anticancer agents such as didemnin B from *Trididemnum solidum*, diazonamide from *Diazona angulata*, and the approved anticancer drug Ecteinascidin **743** (Yondelis™) from *Ecteinascidia turbinata* [107,108].

**Figure 19:**
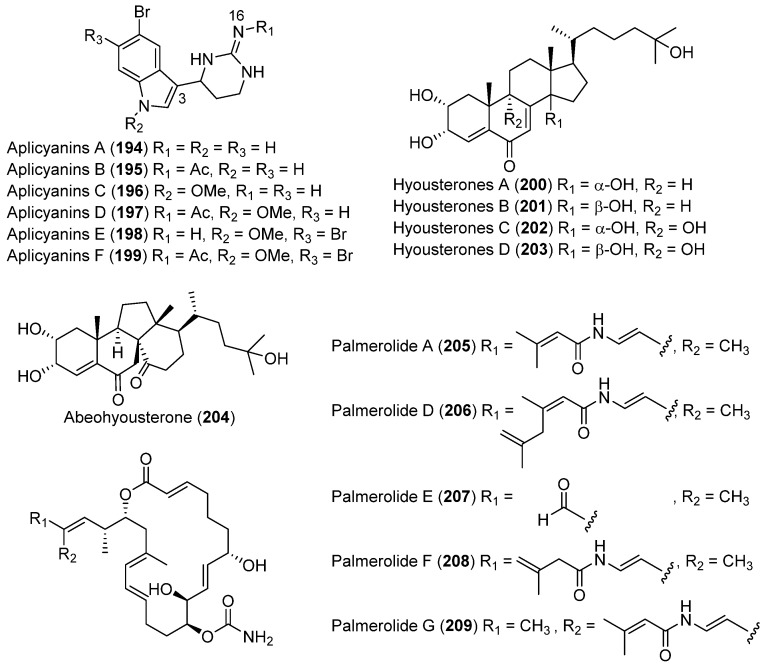
Secondary metabolites derived from the Antarctic tunicates (compounds **194**–**209**).

Bioassay-guided fractionation of CH2Cl2/MeOH extracts of the tunicate *Aplidium cyaneum* collected in Antarctica led to the isolation of aplicyanins A–F (**194**–**199**) (Figure 19), a group of alkaloids containing a bromoindole nucleus and a 6-tetra-hydropyrimidine substituent at C-3. Cytotoxic activity in the submicromolar range as well as antimitotic properties were found for compounds **195**, **197**, and **199**, whereas compounds **194** and **196** proved to be inactive at the highest concentrations tested and compound **198** displayed only mild cytotoxic properties. The results clearly suggested a key role for the presence of the acetyl group at N-16 in the biological activity of this family of compounds [109].

Five new ecdysteroids, hyousterones A–D (**200**–**203**) and abeohyousterone (**204**) (Figure 19), were isolated from the Antarctic tunicate *Synoicum adareanum* by Baker and co-workers. Abeohyousterone (**204**) has moderate cytotoxicity toward several cancer cell lines. Hyousterones bearing the 14β-hydroxy group (**200** and **202**) were weakly cytotoxic, while the 14α-hydroxy hyousterones (**201** and **203**) were devoid of cytotoxicity [110]. Further chemical investigation of the same Antarctic tunicate *S. adareanum* yielded five new bioactive macrolides, palmerolide A (**205**) and D–G (**206**–**209**) (Figure 19). Most of these palmerolides were potent V-ATPase inhibitors and had sub-micromolar activity against melanoma [111,112]. Especially, palmerolide A remained the most potent of this series of natural products against melanoma cells. In the National Cancer Institute (NCI) sixty-cell panel, palmerolide A did not display cytotoxicity below 1 μM against any non-melanoma cell lines. In vivo activity of palmerolide A in mice has been confirmed in the NCI’s hollow fiber assay [113]. Structural features of palmerolide A such as the carbamate and the vinyl amide moieties led the Baker group to hypothesize a bacterial origin for this polyketide. One finding that supported the possibility was the identification of polyketide synthase (PKS) genes, similar to bryostatin biosynthetic genes [114]. Due to promising biological activity and limited access to natural supplies, as well as their challenging structures, palmerolide A and congeneric structures are important targets for chemical synthesis. Recent advances in the synthesis of the palmerolides have been reviewed by Lisboa and Dudley [115]. To date, three total syntheses [116,117,118,119,120] of palmerolide A have been reported. Four groups disclosed formal syntheses [121,122,123,124,125,126], and various synthetic approaches have also been described [127,128,129,130,131,132,133,134,135]. The synthesis of the reported structure of palmerolide C has also been achieved, and a structural revision has been proposed [136].

**Scheme 9:**
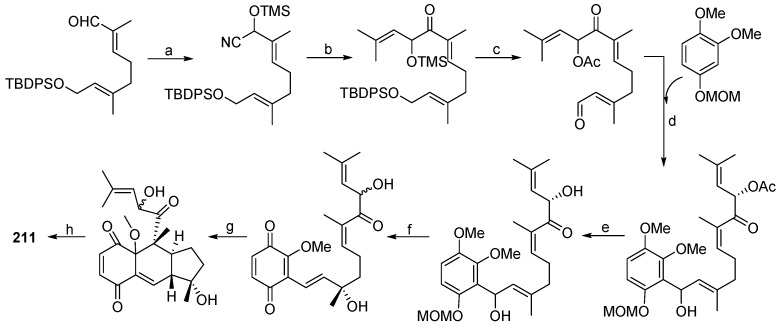
Synthesis of rossinone B (**211**). Reagents and conditions: (**a**) Trimethylsilyl cyanide (TMSCN), NEt_3_, CH_3_CN, 99%; (**b**) LiHMDS, THF, −78 °C, then 3-methyl-2-butenal, TMS migration, 75%; (**c**) 1 N HCl, THF, then Ac_2_O/py, then HF, CH_3_CN, then 2,2-Dimethoxypropane (DMP), 4-(Dicyanomethylene)-2-methyl-6-(4-dimethylaminostyryl)-4*H*-pyran (DCM), 51%; (**d**) *n*BuLi, −78 °C, 88%; (**e**) K_2_CO_3_/MeOH, 79%; (**f**) 6 N HNO_3_, then AgO, 90%; (**g**) toluene, sealed tube, 150 °C; (**h**) CH_3_OH/H_2_O, TsOH, 80 °C, 31%.

**Figure 20:**
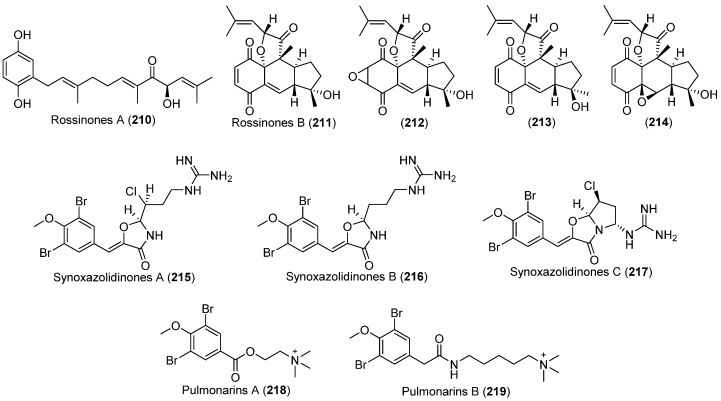
Secondary metabolites derived from the Antarctic tunicates (compounds **210**–**214**) and the Arctic tunicate (compounds **215**–**219**).

In 2009, bioassay-guided fractionation of the Antarctic ascidian *Aplidium* species, led to the characterization of two new biologically active meroterpene derivatives, rossinones A (**210**) and B (**211**) (Figure 20). The rossinones exhibited antiinflammatory, antiviral and antiproliferative activities [137]. In additon, rossinone B (**211**) was proven to take part in the whole-colony chemical defense of *Aplidium falklandicum*, repelling both sea stars and amphipods [138]. The following year, a biomimetic total synthesis of (±)-rossinone B was achieved through a highly efficient strategy as shown in Scheme 9 [139]. Two years later, three novel rossinone-related meroterpenes (**212**–**214**) (Figure 20) were obtained from the Antarctic ascidian *Aplidium fuegiense.* The new compounds were found to be selectively localized in the viscera of the ascidian [140].

From the sub-Arctic ascidian *Synoicum pulmonaria* collected off the Norwegian coast, three new brominated guanidinium oxazolidinones were isolated, named synoxazolidinones A–C (**215**–**217**) (Figure 20) [141,142,143]. The backbone of the compounds contains a 4-oxazolidinone ring rarely seen in natural products. Synoxazolidinones A (**215**) and B (**216**) exhibited antibacterial and antifungal activities, and **215** displayed higher activity than 216 because of the chlorine atom in its structure [141]. Synoxazolidinone C could inhibit the growth of Gram-positive bacteria *Staphylococcus aureus* and methicillin-resistant *S. aureus* at a concentration of 10 μg/mL [142]. In addition, **215** and **217** displayed a broad and high activity toward the adhesion and growth of 16 known biofouling species of marine bacteria, microalgae, and crustaceans. Compound **217** was the most active compound and was comparable to the commercial antifouling product Sea-Nine-211 [144]. Pulmonarins A (**218**) and B (**219**) (Figure 20) were two new dibrominated marine acetylcholinesterase inhibitors that were also isolated from this sub-Arctic ascidian. Both **218** and **219** displayed reversible, noncompetitive acetylcholinesterase inhibition comparable to several known natural acetylcholinesterase inhibitiors [145]. In addition, the pulmonarins were generally active against the adhesion and growth of several bacteria [144].

## 11. Conclusions

As demonstrated by this review, polar organisms have yielded an impressive array of novel compounds (Table 1) with complex structures and potent biological activities including the cytotoxic cyclic acylpeptides, mixirins A–C (**1**–**3**), from the Arctic marine bacterium *Bacillus* sp. [15]; the unusual antibacterial polyketide, lindgomycin (**46**) from the culture broth of a Lindgomycetaceae strain [36]; the antioxidant compound ramalin (**104**) from the Antarctic lichen *Ramalina terebrata* [61]; the new antiparasitic tricyclic sesquiterpenoids, shagenes A (**130**) and B (**131**) from an undescribed Antarctic soft coral [76]; and the new macrolides, palmerolide A (**205**) and D–G (**206**–**209**) with potent V-ATPase inhibitory and sub-micromolar activity against melanoma from the Antarctic tunicate *Synoicum adareanum* [111,112].

The natural products derived from polar regions appear to have a high hit rate regarding biological activity, varying from cytotoxic, enzyme inhibitory, antioxidant, antiparasitic, antiviral to antibacterial and so on. Secondary metabolism in polar habitats is largely driven by ecological requirements of the producing organism. Successful organisms will often have specific metabolic pathways that produce unique functional natural products that bestow ecological advantage, increasing the possibility of finding pharmaceutical lead molecules.

However, when compared to the large number of polar microorganisms which have been reported, very few have been screened for the production of interesting secondary metabolites. This situation may be attributed to the difficulties in cultivating polar microorganisms, some of which cannot survive under normal laboratory conditions and therefore cannot be cultured using traditional techniques. As yet, the potential of this area remains virtually untapped. Nowadays, advances in laboratory techniques have led to cultivation of some previously inaccessible extremophiles. Moreover, new tools developed recently in the fields of bioinformatics [146], analytics [147], and molecular biology [148], in combination with rapid improvement in sequencing technology, might herald a new era of research into this specific source.

## Figures and Tables

**Table 1:** Novel natural products isolated from polar organisms.

PHYLUM/Class	Species	Compounds	Bioactivity	Region	References
MICROORGANISMS					
Bacteria	*Bacillus* sp.	**1**–**3**	Cytotoxic	Arctic	[15]
	*Salegentibacter* sp.	**4**–**14**	Antimicrobial and cytotoxic	Arctic	[16,17]
	*Pseudoalteromonas haloplanktis*	**15 16**, **17**	Rradical scavenging	Antarctic	[18]
	*Nostoc* sp.	**18**	Antibacterial	Antarctic	[19]
	*Janthinobacterium* sp.	**19**, **20**	Antimycobacterial	Antarctic	[20]
	*Pseudomonas* sp.	**21**, **22**	Antibacterial	Antarctic	[21]
Actinomyces	*Streptomyces* sp.	**23**–**25**		Arctic	[22]
	*Nocardiopsis* sp.	**26**	Anti-angiogenesis	Arctic	[23]
	*Nocardia dassonvillei*	**27**	Antifungal and cytotoxic	Arctic	[24]
	*Streptomyces nitrosporeus*	**28**, **29**	Antiviral	Arctic	[25]
	*Streptomyces* sp.	**30**	Enzyme inhibitory	Arctic	[27]
		**31**	Enzyme inhibitory and cytotoxic		
	*Streptomyces griseus*	**32**	Antibacterial	Antarctic	[28]
	*Streptomyces griseus*	**33**	Neuroprotective	Antarctic	[29]
	*Microbispora aerata*	**34**	Antiproliferative and cytotoxic	Antarctic	[30]
	*Nocardiopsis* sp.	**35**, **36**		Antarctic	[31]
Fungi	*Penicillium algidum*	**37**	Anticancer	Arctic	[32]
	*Eutypella* sp.	**38**–**40**	Cytotoxicity	Arctic	[33,34,35]
		**41**	Antibacterial		
		**42**	Cytotoxicity		
		**43**–**45**	Immunosuppression		
	Lindgomycetaceae	**46**, **47**	Antibacterial	Arctic	[36,37]
	*Trichoderma polysporum*	**48**	Antifungal	Arctic	[38]
	*Geomyces* sp.	**49**, **50**		Antarctic	[39]
		**51**–**53**	Antifungal and antibacterial		
	*Trichoderma asperellum*	**54**–**59**	Antifungal	Antarctic	[40]
	*Oidiodendron truncatum*	**60**–**62**	Cytotoxic	Antarctic	[42]
		**63**–**66**			
	*Penicillium crustosu m*	**67**, **68**	NF-κB inhibitory	Antarctic	[43]
	*Penicillium* sp.	**69**	Moderate cytotoxic	Antarctic	[45,46]
		**70**–**78**			
		**79**	Cytotoxic		
	*Penicillium funiculosum*	**80**, **81**		Antarctic	[47]
	*Pseudogymnoascus* sp.	**82**–**85**		Antarctic	[48]
	*Aspergillus ochraceopetaliformis*	**86**–**90**	Antiviral	Antarctic	[49]
		**91**			
LICHEN	*Stereocaulon alpinum*	**92**	Cytotoxic and enzyme inhibitory	Antarctic	[55,56,57,58,59]
		**93**–**98**	Enzyme inhibitory		
		**99**	Antioxidant and antibacterial		
	*Huea* sp.	**100**–**103**	Enzyme inhibitory	Antarctic	[60]
	*Ramalina terebrata*	**104**	Antioxidant	Antarctic	[61]
MOSS	*Polytrichastrum alpinum*	**105**, **106**	Enzyme inhibitory	Antarctic	[62]
BRYOZOANS	*Tegella* cf. *spitzbergensis*	**107**–**110**	Antibacterial	Arctic	[64]
CNIDARIANS	*Ainigmaptilon antarcticus*	**111**, **112**	Predation inhibitory	Antarctic	[66]
	*Dasystenella acanthina*	**113**	Ichthyotoxic	Antarctic	[67]
	*Anthomastus bathyproctus*	**114**–**120**	Weak cytotoxic	Antarctic	[68]
	*Alcyonium grandis*	**121**–**129**		Antarctic	[69]
	Undescribed octocoral	**130**, **131**	Antiparasitic	Antarctic	[76]
	*Thuiria breitfussi*	**132**, **133**		Arctic	[77]
	*Gersemia fruticosa*	**134**–**136**		Arctic	[80]
		**137**	Antibacterial		
ECHINODERMS	*Staurocucumis liouvillei*	**138**, **139**	Antiviral	Antarctic	[81]
	*Staurocucumis turqueti*	**140**		Antarctic	[82]
	*Achlionice violaecuspidata*	**141**–**143**		Antarctic	[83]
MOLLUSCS	*Austrodoris kerguelenensis*	**144**–**147**		Antarctic	[84,85]
	*Austrodoris* *kerguelenensis*	**148**–**166**	Erythroleukemia inhibitory	Antarctic	[89]
	*Charcotia granulosa*	**167**		Antarctic	[91]
SPONGES	*Haliclona viscosa*	**168**–**173**		Arctic	[93,94,95,96]
	*Suberites caminatus*	**174**–**176**		Antarctic	[99,100]
	*Dendrilla membranosa*	**177**–**180**		Antarctic	[101]
	*Suberites* sp.	**181**–**183**		Antarctic	[102]
	*Crella* sp.	**184**–**188**	Antibacterial and antifungal	Antarctic	[103]
	*Dendrilla membranosa*	**189**	Antibacterial	Antarctic	104]
	*Geodia barretti*	**190**	Enzyme inhibitory	Arctic	[105]
		**191**–**193**			
TUNICATES	*Aplidium cyaneum*	**194**–**199**	Antimitotic	Antarctic	[109]
	*Synoicum adareanum*	**200**–**203**	Moderate cytotoxic	Antarctic	[110,111,112]
		**204**			
		**205**–**209**	Cytotoxic and enzyme inhibitory		
	*Aplidium* sp.	**210**, **211**	Antiinflammatory, antiviral and antiproliferative	Antarctic	[137]
	*Aplidium fuegiense*	**212**–**214**		Antarctic	[140]
	*Synoicum pulmonaria*	**215**–**217**	Antibacterial and antifungal	Arctic	[141,142,143,144,145]
		**218**, **219**	Enzyme inhibitory and antibacterial		
